# *N*,*N*-Dimethyl-3-(1-naphth­yloxy)-3-(2-thien­yl)propan-1-amine

**DOI:** 10.1107/S1600536808003255

**Published:** 2008-02-06

**Authors:** Xiao Tao, Xiao-Qing Zhang, Lin Yuan, Jin-Tang Wang

**Affiliations:** a Department of Applied Chemistry, College of Science, Nanjing University of Technology, Nanjing 210009, People’s Republic of China; b Nanjing Huawei Medicinal Science, Development Co., Ltd., Nanjing 210036, People’s Republic of China

## Abstract

The title compound, C_19_H_21_NOS, is an inter­mediate for the synthesis of duloxetine hydro­chloride. In the mol­ecular structure, the thio­phene and naphthalene ring systems make a dihedral angle of 87.5°. All bond lengths and angles involving heteroatoms are as expected. In the crystal structure, no classical hydrogen bonds are found.

## Related literature

For the preparation of duloxetine see: Deeter *et al.* (1990 ▶). For related hydroxy derivatives of the title mol­ecule, see: Tao, Bin *et al.* (2006 ▶); Tao, Li *et al.* (2006 ▶).
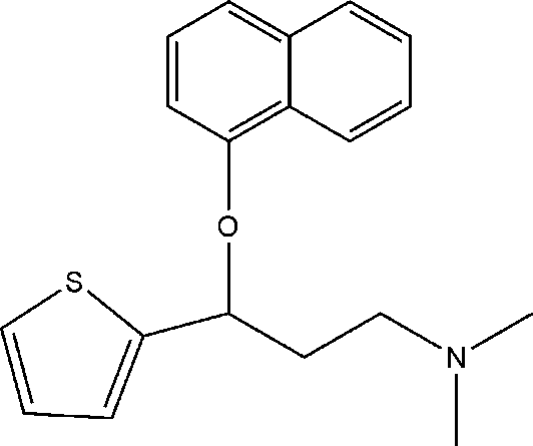


## Experimental

### 

#### Crystal data

C_19_H_21_NOS
*M* *_r_* = 311.43
Monoclinic, 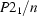

*a* = 9.6140 (19) Å
*b* = 18.578 (4) Å
*c* = 9.905 (2) Å
β = 104.53 (3)°
*V* = 1712.5 (6) Å^3^
*Z* = 4
Mo *K*α radiation
μ = 0.19 mm^−1^
*T* = 293 (2) K
0.40 × 0.30 × 0.10 mm

#### Data collection

Enraf–Nonius CAD-4 diffractometer
Absorption correction: ψ scan (North *et al.*, 1968 ▶) *T*_min_ = 0.928, *T*_max_ = 0.981
3550 measured reflections
3352 independent reflections
2009 reflections with *I* > 2s(*I*)
*R*_int_ = 0.038
3 standard reflections every 200 reflections intensity decay: <1%

#### Refinement

*R*[*F* ^2^ > 2σ(*F* ^2^)] = 0.070
*wR*(*F* ^2^) = 0.194
*S* = 1.04
3352 reflections
199 parameters
H-atom parameters constrained
Δρ_max_ = 0.45 e Å^−3^
Δρ_min_ = −0.32 e Å^−3^

### 

Data collection: *CAD-4 Software* (Enraf–Nonius, 1981 ▶); cell refinement: *CAD-4 Software*; data reduction: *XCAD4* (Harms & Wocadlo, 1995 ▶); program(s) used to solve structure: *SHELXS97* (Sheldrick, 2008 ▶); program(s) used to refine structure: *SHELXL97* (Sheldrick, 2008 ▶); molecular graphics: *SHELXTL* (Sheldrick, 2008 ▶); software used to prepare material for publication: *SHELXTL*.

## Supplementary Material

Crystal structure: contains datablocks I, global. DOI: 10.1107/S1600536808003255/bh2157sup1.cif

Structure factors: contains datablocks I. DOI: 10.1107/S1600536808003255/bh2157Isup2.hkl

Additional supplementary materials:  crystallographic information; 3D view; checkCIF report

## Figures and Tables

**Fig. 1.**
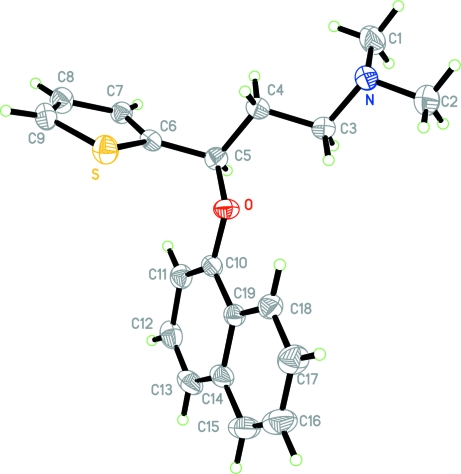
The structure of (I). Displacement ellipsoids are drawn at the 30% probability level.

**Fig. 2.**
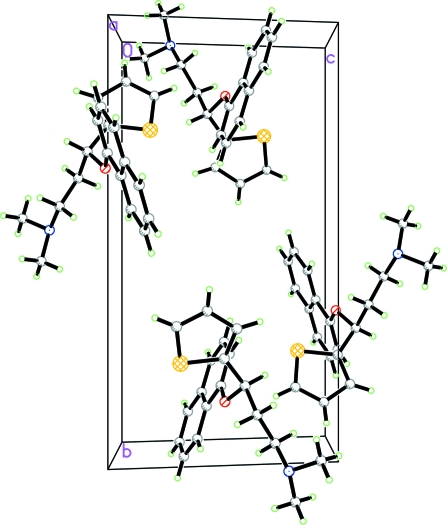
The crystal packing of (I) viewed along [100].

## supplementary crystallographic information

### Comment

The title compound, (I), an is intermediate for Duloxetine hydrochloride (Deeter *et al.*, 1990). The crystal structure determination of (I) has been carried out in order to elucidate its molecular conformation. In the molecular structure (Fig. 1) bond lengths and angles are within normal ranges and compare well with those observed in the corresponding alcohol, 3-hydroxy-*N*,*N*-dimethyl-3-(2-thienyl)propanamine (Tao, Bin *et al.*, 2006; Tao, Li *et al.*, 2006). The thiophene (S/C6···C9) and naphthalene (C10···C19) rings are planar and the dihedral angle between them is 87.5°. In the crystal structure, no classic hydrogen bonds are found. It may then be assumed that dipole-dipole and van der Waals interactions are effective for the molecular packing (Fig. 2).

### Experimental

*N*,*N*-Dimethyl-3-(2-thienyl)-3-hydroxylpropanamine (9.25 g, 0.05 mol) was dissolved in 30 ml of dimethylsulfoxide. Sodium hydride (60%, 1.5 g, 0.225 mol) was added to the solution with stirring at room temperature for another 15 min. Then, 1-fluoronaphthalene (8.75 g, 0.06 mol) was added, and the mixture was stirred for 8 h. at 323 K. The mixture was poured into 50 ml of ice water, and the pH was adjusted to 4–5 using acetic acid. 50 ml of hexane was added, stirred and the layers were separated. The aqueous phase was stirred with 30 ml of hexane, the pH was adjusted to 12 using 25% aqueous sodium hydroxide, 30 ml of ethyl acetate was added, stirred and the layers were separated. The aqueous phase was extracted with another 30 ml of ethyl acetate, and the organic extracts were combined, washed with 30 ml of water, dried over magnesium sulfate. The solvent was removed under vacuum to obtain (I) as a brown oil (yield: 11.3 g, 72.9%). The title compound (I) was dissolved in a mixture of ethanol and acetone (2:1). After 14 days, brown single crystals were collected.

### Refinement

All H atoms were included in the riding model approximation with C—H distances constrained to 0.93 (aromatic CH) 0.96 (methyl CH_3_), 0.97 (methylene CH_2_) and 0.98 Å (methine CH), and with *U*_iso_(H) = 1.5 *U*_eq_(carrier C) for the methyl groups and *U*_iso_(H) = 1.2 *U*_eq_(carrier C) otherwise.

### Crystal data

**Table:**

C_19_H_21_NOS	*F*_000_ = 664
*M**_r_* = 311.43	*D*_x_ = 1.208 Mg m^−^^3^
Monoclinic, *P*2_1_/*n*	Melting point = 386–388 K
Hall symbol: -P 2yn	Mo *K*α radiation λ = 0.71073 Å
*a* = 9.6140 (19) Å	Cell parameters from 25 reflections
*b* = 18.578 (4) Å	θ = 10–13º
*c* = 9.905 (2) Å	µ = 0.19 mm^−^^1^
β = 104.53 (3)º	*T* = 293 (2) K
*V* = 1712.5 (6) Å^3^	Block, brown
*Z* = 4	0.40 × 0.30 × 0.10 mm

### Data collection

**Table:**

Enraf–Nonius CAD-4 diffractometer	*R*_int_ = 0.038
Radiation source: fine-focus sealed tube	θ_max_ = 26.0º
Monochromator: graphite	θ_min_ = 2.4º
*T* = 293(2) K	*h* = 0→11
ω/2θ scans	*k* = 0→22
Absorption correction: ψ scan(North *et al.*, 1968)	*l* = −12→11
*T*_min_ = 0.928, *T*_max_ = 0.981	3 standard reflections
3550 measured reflections	every 200 reflections
3352 independent reflections	intensity decay: <1%
2009 reflections with *I* > 2s(*I*)	

### Refinement

**Table:**

Refinement on *F*^2^	Secondary atom site location: difference Fourier map
Least-squares matrix: full	Hydrogen site location: inferred from neighbouring sites
*R*[*F*^2^ > 2σ(*F*^2^)] = 0.070	H-atom parameters constrained
*wR*(*F*^2^) = 0.194	*w* = 1/[σ^2^(*F*_o_^2^) + (0.08*P*)^2^ + 0.85*P*] where *P* = (*F*_o_^2^ + 2*F*_c_^2^)/3
*S* = 1.04	(Δ/σ)_max_ < 0.001
3352 reflections	Δρ_max_ = 0.45 e Å^−^^3^
199 parameters	Δρ_min_ = −0.32 e Å^−^^3^
Primary atom site location: structure-invariant direct methods	Extinction correction: none

### Fractional atomic coordinates and isotropic or equivalent isotropic displacement parameters (Å^2^)

**Table:**

	*x*	*y*	*z*	*U*_iso_*/*U*_eq_	
S	0.03342 (12)	0.25309 (6)	0.17997 (10)	0.0561 (3)	
O	0.1693 (2)	0.34125 (12)	0.0007 (2)	0.0445 (6)	
N	−0.1370 (3)	0.47356 (16)	−0.2560 (3)	0.0517 (8)	
C1	−0.2401 (5)	0.4470 (2)	−0.3807 (5)	0.0777 (14)	
H1A	−0.2777	0.4015	−0.3607	0.117*	
H1B	−0.1929	0.4412	−0.4547	0.117*	
H1C	−0.3173	0.4809	−0.4086	0.117*	
C2	−0.0803 (5)	0.5421 (2)	−0.2843 (5)	0.0753 (13)	
H2A	−0.0133	0.5593	−0.2016	0.113*	
H2B	−0.1575	0.5760	−0.3123	0.113*	
H2C	−0.0322	0.5367	−0.3577	0.113*	
C3	−0.0227 (4)	0.4218 (2)	−0.2029 (4)	0.0517 (10)	
H3A	0.0559	0.4461	−0.1383	0.062*	
H3B	0.0131	0.4041	−0.2800	0.062*	
C4	−0.0708 (4)	0.3584 (2)	−0.1295 (4)	0.0506 (10)	
H4A	−0.1016	0.3758	−0.0493	0.061*	
H4B	−0.1527	0.3357	−0.1924	0.061*	
C5	0.0463 (4)	0.30309 (19)	−0.0816 (4)	0.0439 (9)	
H5A	0.0711	0.2822	−0.1634	0.053*	
C6	0.0029 (4)	0.24339 (18)	0.0033 (4)	0.0406 (8)	
C7	−0.0719 (4)	0.17959 (18)	−0.0465 (4)	0.043	
H7A	−0.0997	0.1649	−0.1392	0.052*	
C8	−0.0976 (4)	0.1414 (2)	0.0708 (4)	0.0542 (10)	
H8A	−0.1447	0.0973	0.0620	0.065*	
C9	−0.0482 (4)	0.1743 (2)	0.1943 (4)	0.0510 (10)	
H9A	−0.0580	0.1556	0.2785	0.061*	
C10	0.3039 (4)	0.31259 (19)	0.0180 (3)	0.0403 (8)	
C11	0.3328 (4)	0.2446 (2)	−0.0182 (4)	0.0498 (9)	
H11A	0.2584	0.2131	−0.0572	0.060*	
C12	0.4781 (5)	0.2225 (2)	0.0045 (4)	0.0574 (11)	
H12A	0.4982	0.1762	−0.0211	0.069*	
C13	0.5882 (5)	0.2673 (2)	0.0627 (4)	0.0603 (11)	
H13A	0.6825	0.2515	0.0761	0.072*	
C14	0.5612 (4)	0.3372 (2)	0.1027 (4)	0.0496 (10)	
C15	0.6714 (4)	0.3854 (3)	0.1646 (4)	0.0677 (13)	
H15A	0.7665	0.3704	0.1814	0.081*	
C16	0.6431 (5)	0.4528 (3)	0.2002 (5)	0.0746 (13)	
H16A	0.7185	0.4836	0.2397	0.089*	
C17	0.5021 (5)	0.4765 (2)	0.1781 (4)	0.0655 (12)	
H17A	0.4837	0.5230	0.2040	0.079*	
C18	0.3900 (4)	0.4320 (2)	0.1186 (4)	0.0481 (9)	
H18A	0.2961	0.4485	0.1038	0.058*	
C19	0.4161 (4)	0.36147 (19)	0.0798 (3)	0.0409 (8)	

### Atomic displacement parameters (Å^2^)

**Table:**

	*U*^11^	*U*^22^	*U*^33^	*U*^12^	*U*^13^	*U*^23^
S	0.0651 (7)	0.0585 (6)	0.0425 (5)	−0.0028 (5)	0.0094 (5)	−0.0023 (5)
O	0.0335 (13)	0.0410 (14)	0.0566 (15)	−0.0007 (11)	0.0070 (11)	−0.0068 (11)
N	0.0482 (19)	0.0464 (18)	0.058 (2)	0.0008 (15)	0.0083 (15)	0.0088 (15)
C1	0.065 (3)	0.070 (3)	0.081 (3)	−0.003 (2)	−0.011 (2)	0.018 (3)
C2	0.074 (3)	0.054 (3)	0.095 (4)	−0.003 (2)	0.017 (3)	0.013 (3)
C3	0.041 (2)	0.055 (2)	0.057 (2)	−0.0018 (18)	0.0077 (18)	0.0090 (19)
C4	0.040 (2)	0.055 (2)	0.053 (2)	0.0009 (18)	0.0033 (17)	0.0086 (19)
C5	0.044 (2)	0.045 (2)	0.0414 (19)	−0.0021 (17)	0.0088 (16)	−0.0027 (16)
C6	0.0393 (19)	0.044 (2)	0.0367 (17)	0.0071 (16)	0.0068 (14)	0.0008 (16)
C7	0.043	0.043	0.043	0.000	0.011	0.000
C8	0.060 (3)	0.041 (2)	0.059 (3)	−0.0030 (19)	0.012 (2)	−0.0004 (19)
C9	0.062 (3)	0.047 (2)	0.046 (2)	0.0077 (19)	0.0170 (19)	0.0069 (18)
C10	0.0380 (19)	0.048 (2)	0.0372 (19)	0.0080 (16)	0.0127 (15)	0.0072 (16)
C11	0.055 (2)	0.050 (2)	0.048 (2)	0.0039 (19)	0.0182 (18)	0.0050 (19)
C12	0.069 (3)	0.056 (2)	0.055 (2)	0.022 (2)	0.029 (2)	0.011 (2)
C13	0.050 (2)	0.084 (3)	0.049 (2)	0.025 (2)	0.0167 (19)	0.016 (2)
C14	0.041 (2)	0.070 (3)	0.039 (2)	0.0091 (19)	0.0118 (17)	0.0115 (19)
C15	0.039 (2)	0.103 (4)	0.059 (3)	−0.002 (2)	0.008 (2)	0.005 (3)
C16	0.051 (3)	0.101 (4)	0.067 (3)	−0.019 (3)	0.004 (2)	−0.007 (3)
C17	0.064 (3)	0.060 (3)	0.069 (3)	−0.009 (2)	0.011 (2)	−0.010 (2)
C18	0.046 (2)	0.052 (2)	0.046 (2)	−0.0025 (18)	0.0120 (17)	0.0017 (18)
C19	0.0399 (19)	0.050 (2)	0.0324 (18)	0.0023 (17)	0.0085 (15)	0.0062 (16)

### Geometric parameters (Å, °)

**Table:**

S—C6	1.709 (3)	C7—H7A	0.9300
S—C9	1.683 (4)	C8—C9	1.343 (5)
O—C10	1.370 (4)	C8—H8A	0.9300
O—C5	1.443 (4)	C9—H9A	0.9300
N—C2	1.440 (5)	C10—C11	1.360 (5)
N—C3	1.455 (5)	C10—C19	1.425 (5)
N—C1	1.462 (5)	C11—C12	1.419 (5)
C1—H1A	0.9600	C11—H11A	0.9300
C1—H1B	0.9600	C12—C13	1.357 (6)
C1—H1C	0.9600	C12—H12A	0.9300
C2—H2A	0.9600	C13—C14	1.400 (5)
C2—H2B	0.9600	C13—H13A	0.9300
C2—H2C	0.9600	C14—C15	1.406 (6)
C3—C4	1.517 (5)	C14—C19	1.429 (5)
C3—H3A	0.9700	C15—C16	1.345 (6)
C3—H3B	0.9700	C15—H15A	0.9300
C4—C5	1.511 (5)	C16—C17	1.390 (6)
C4—H4A	0.9700	C16—H16A	0.9300
C4—H4B	0.9700	C17—C18	1.369 (5)
C5—C6	1.513 (5)	C17—H17A	0.9300
C5—H5A	0.9800	C18—C19	1.405 (5)
C6—C7	1.410 (5)	C18—H18A	0.9300
C7—C8	1.435 (5)		
			
C9—S—C6	91.74 (18)	C6—C7—H7A	126.0
C10—O—C5	119.6 (3)	C8—C7—H7A	126.0
C2—N—C3	111.4 (3)	C9—C8—C7	114.8 (3)
C2—N—C1	110.1 (3)	C9—C8—H8A	122.6
C3—N—C1	111.9 (3)	C7—C8—H8A	122.6
N—C1—H1A	109.5	C8—C9—S	112.6 (3)
N—C1—H1B	109.5	C8—C9—H9A	123.7
H1A—C1—H1B	109.5	S—C9—H9A	123.7
N—C1—H1C	109.5	C11—C10—O	125.2 (3)
H1A—C1—H1C	109.5	C11—C10—C19	121.4 (3)
H1B—C1—H1C	109.5	O—C10—C19	113.3 (3)
N—C2—H2A	109.5	C10—C11—C12	119.1 (4)
N—C2—H2B	109.5	C10—C11—H11A	120.5
H2A—C2—H2B	109.5	C12—C11—H11A	120.5
N—C2—H2C	109.5	C13—C12—C11	121.4 (4)
H2A—C2—H2C	109.5	C13—C12—H12A	119.3
H2B—C2—H2C	109.5	C11—C12—H12A	119.3
N—C3—C4	113.1 (3)	C12—C13—C14	120.6 (4)
N—C3—H3A	109.0	C12—C13—H13A	119.7
C4—C3—H3A	109.0	C14—C13—H13A	119.7
N—C3—H3B	109.0	C13—C14—C15	122.7 (4)
C4—C3—H3B	109.0	C13—C14—C19	119.4 (4)
H3A—C3—H3B	107.8	C15—C14—C19	117.8 (4)
C5—C4—C3	112.7 (3)	C16—C15—C14	121.8 (4)
C5—C4—H4A	109.1	C16—C15—H15A	119.1
C3—C4—H4A	109.1	C14—C15—H15A	119.1
C5—C4—H4B	109.1	C15—C16—C17	120.5 (4)
C3—C4—H4B	109.1	C15—C16—H16A	119.8
H4A—C4—H4B	107.8	C17—C16—H16A	119.8
O—C5—C4	106.5 (3)	C18—C17—C16	120.5 (4)
O—C5—C6	110.3 (3)	C18—C17—H17A	119.8
C4—C5—C6	112.7 (3)	C16—C17—H17A	119.8
O—C5—H5A	109.1	C17—C18—C19	120.4 (4)
C4—C5—H5A	109.1	C17—C18—H18A	119.8
C6—C5—H5A	109.1	C19—C18—H18A	119.8
C7—C6—C5	127.5 (3)	C18—C19—C10	122.9 (3)
C7—C6—S	112.9 (3)	C18—C19—C14	119.0 (3)
C5—C6—S	119.4 (3)	C10—C19—C14	118.1 (3)
C6—C7—C8	108.0 (3)		
			
C2—N—C3—C4	161.7 (4)	C19—C10—C11—C12	−0.9 (5)
C1—N—C3—C4	−74.6 (4)	C10—C11—C12—C13	0.7 (6)
N—C3—C4—C5	176.9 (3)	C11—C12—C13—C14	0.2 (6)
C10—O—C5—C4	−157.5 (3)	C12—C13—C14—C15	179.4 (4)
C10—O—C5—C6	80.0 (4)	C12—C13—C14—C19	−1.0 (6)
C3—C4—C5—O	53.6 (4)	C13—C14—C15—C16	178.9 (4)
C3—C4—C5—C6	174.6 (3)	C19—C14—C15—C16	−0.8 (6)
O—C5—C6—C7	−154.8 (3)	C14—C15—C16—C17	0.9 (7)
C4—C5—C6—C7	86.3 (4)	C15—C16—C17—C18	−0.7 (7)
O—C5—C6—S	30.0 (4)	C16—C17—C18—C19	0.4 (6)
C4—C5—C6—S	−88.8 (3)	C17—C18—C19—C10	179.7 (3)
C9—S—C6—C7	0.6 (3)	C17—C18—C19—C14	−0.3 (5)
C9—S—C6—C5	176.4 (3)	C11—C10—C19—C18	−179.9 (3)
C5—C6—C7—C8	−176.3 (3)	O—C10—C19—C18	−0.1 (5)
S—C6—C7—C8	−0.8 (4)	C11—C10—C19—C14	0.2 (5)
C6—C7—C8—C9	0.7 (5)	O—C10—C19—C14	180.0 (3)
C7—C8—C9—S	−0.3 (4)	C13—C14—C19—C18	−179.2 (3)
C6—S—C9—C8	−0.2 (3)	C15—C14—C19—C18	0.5 (5)
C5—O—C10—C11	−10.8 (5)	C13—C14—C19—C10	0.8 (5)
C5—O—C10—C19	169.4 (3)	C15—C14—C19—C10	−179.6 (3)
O—C10—C11—C12	179.3 (3)		
